# Some Older London Hospitals: How They Have Been Re-Created

**Published:** 1915-12-18

**Authors:** 


### SOME OLDER LONDON HOSPITALS.

### How They Have Been Re-created.

For forty-five years it has been our privilege to take an active part in the evolution of the modern hospital, its organisation, construction, and development. As the years have passed we have enjoyed the advantage of possessing the confidence of most hospital workers and of many of the representative and largest givers to the voluntary hospitals. Not once, but often, when occasion has arisen for the planning and erection of a new hospital, either the capitalist who was mainly responsible for financing the venture or the architects who were commissioned to prepare the plans or the representatives of the administrative or medical staff, one or all, have consulted the writer. Many years' residence in and the responsible charge of various types of voluntary hospitals, the close inspection at regular intervals of practically all the principal hospitals in Europe, the United States of America, and other countries, and a continuous study of hospitals and their work, virtually without intermission for forty-five years, made the planning and erection of the new King's College Hospital buildings at Denmark Hill a matter of deep interest and one which filled us with hope. We realised that too often new hospital buildings in the past had been merely reproductions of existing buildings with the addition of some new features, without any real attempt to secure originality and up-to-dateness based upon modern scientific developments.

### Causes of Disappointment.

Too often a free hand to those responsible, owing to abundance of funds, has resulted in justifiable disappointment to hospital experts. They, we know, share with the writer the knowledge that before the institution of the King's Hospital Fund, when it was manifest that the condition of a considerable number of the larger hospitals in the Metropolis made it essential that they should be entirely reconstructed or rebuilt, there was not in fact any relatively new hospital of the first importance which could be accepted without question as perfect and up to date.

### The King's Fund's Influence.

The institution of the King Edward VII. Hospital Fund has done much to remove anomalies and raise the status of the voluntary hospitals. One of its first results to all the London hospitals was to extend to them the driving force created by King Edward's interest in our hospitals, and the exercise of his influence on their behalf. These forces had proved fruitful in securing the adoption of a scheme which has led to the re-creation and to the re-establishment of the financial strength of Guy's Hospital, of which King Edward, then Prince of Wales, accepted the presidency in 1896. The first-fruits of his Fund in the way of reconstruction and financial strength was the commencement of the rebuilding of the London Hospital at Whitechapel, owing to the encouragement extended to the authorities there in 1897 by the Prince of Wales's Hospital Fund, as it was then called. Since then the King's Fund has systematically and most materially aided a number of the metropolitan hospitals in the same direction, with results which have won for the voluntary system an ever-increasing volume of support in money and personal service for the hospitals in the Metropolis of the Empire. We think it is essential to make these points in our present Special Number, the aim of which throughout is to encourage all hospital authorities to increase their confidence in the voluntary system, to promote its extension and strengthen its foundations in all directions after the conclusion of the great war.

### Guy's and the London Hospital.

The reconstructions of Guy's Hospital and of the London Hospital, especially, have been carried out with such thoroughness and admirable determination that those in search of the later developments in hospital construction and of the best instances of up-to-date units with the efficient organisation of new departments of hospital work may usefully make it their first business to study in detail these institutions as they exist to-day, for they will find them fruitful fields of knowledge and helpfulness. These reconstructions have been carried through with ability and conscientious zeal, which have made for notable success in many directions.

